# Association Between Lipid Profile Measurements and Mortality Outcomes Among Older Adults in a Primary Care Setting: A Retrospective Cohort Study

**DOI:** 10.7759/cureus.35087

**Published:** 2023-02-16

**Authors:** Qusay F Almahmoud, Saud M Alhaidar, Abdullah H Alkhenizan, Loay K Basudan, Mohammed Shafiq

**Affiliations:** 1 Family Medicine, King Faisal Specialist Hospital and Research Centre, Riyadh, SAU

**Keywords:** hdl cholesterol, cardiovascular outcomes, triglycerides, total cholesterol, ldl cholesterol, older adults, all-cause mortality, cholesterol level

## Abstract

Background

Lipid profile components play a role in predicting the development of cardiovascular disease and hence mortality, but recent studies have shown mixed results in the older population. The aim of our study was to investigate the association between levels of lipid profile components with all-cause mortality and cardiovascular outcomes among older adults in a primary care setting in Riyadh, Saudi Arabia.

Methods

A retrospective cohort study was performed among 485 individuals aged 60 years and older who visited the family medicine clinics linked to a tertiary care hospital during the first six months of 2010. The electronic charts of the participants were reviewed up to April 2022 to gather relevant data. Each lipid profile component, including total cholesterol (TC), low-density lipoprotein cholesterol (LDL-C), high-density lipoprotein cholesterol (HDL-C), and triglycerides (TGs), was categorized into four quartiles. LDL was calculated using the Friedewald formula. Cardiovascular outcomes included ischemic heart disease (IHD), heart failure (HF), and stroke.

Results

The mean follow-up period was 12 years. The elderly participants with the lowest HDL-C quartile (<1.1 mmol/L) were at higher risk of all-cause mortality (adjusted hazard ratio of 2.023 (95% CI 1.21-3.38)) and IHD (adjusted hazard ratio 3.2 (95% CI 1.6-6.2)). High TC (≥5.7 mmol/L) was associated with an increased risk of HF (adjusted hazard ratio 2.1 (95% CI 1.1-4.0)).

Conclusion

In patients aged 60 years and older, low HDL-C (<1.1 mmol/L) was associated with a higher risk for all-cause mortality and IHD, and high TC was associated with an increased risk of having HF. No significant association was found for LDL-C, TC, and TGs with all-cause mortality.

## Introduction

The impact of elevated total cholesterol (TC) and low-density lipoprotein cholesterol (LDL-C) on cardiovascular and all-cause mortality in the general population is well established [1,2]. However, this association seems to be more complex in older individuals as multiple studies have shown that elevated TC is not associated or even inversely associated with mortality in the elderly [3-5]. Similarly, with LDL-C, the results are inconsistent, with some studies showing an inverse association with all-cause mortality [6,7].

High-density lipoprotein cholesterol (HDL-C) has a well-known role as the “good” cholesterol in protecting against cardiovascular and all-cause mortality, but in the older population, there are different results, with studies showing that only the lowest levels are associated with the highest mortality [8,9], while other studies show a U-shaped relationship with the lowest and highest quartiles corresponding with the highest mortality [10,11]. For triglycerides (TGs), a meta-analysis study showed that elevated levels were associated with a higher risk for cardiovascular and all-cause mortality [12], although some studies showed that the relationship weakens with older age [13,14]. 

Cardiovascular disease (CVD) is the most common cause of death globally according to the World Health Organization [15], and one of the most important risk factors for CVD is dyslipidemia [16]. Studies involving the older population found that low HDL-C was significantly associated with an increased risk for ischemic heart disease (IHD) and stroke [9,17]. For LDL-C, some studies show a higher risk for CVD with high levels [18,19], while other studies show no association [20,21]. Moreover, a study reviewing the association between high TC and CVD in the elderly found a strong association with IHD [22]. For TGs, only a few studies have targeted the elderly population and found that higher levels are associated with increased CVD risk [23,24]. No regional or local studies are available in the literature on the association between lipid profile measurements and mortality in the older population. This study investigates the association of plasma lipid profile concentrations with all-cause mortality and cardiovascular outcomes among older adults (60 years of age and older).

## Materials and methods

We performed a retrospective cohort study using electronic health records from King Faisal Specialist Hospital and Research Centre (KFSH&RC). All patients aged 60 years and older who presented to the family medicine clinic in KFSH&RC during the period from January 1, 2010, to June 30, 2010, and underwent lipid profile blood tests were enrolled. We excluded patients who died within one year after the blood test or those who were lost to follow-up. Demographic data collected included age and gender. 

Lipid profile levels

The lipid profile included TC, LDL-C, HDL-C, and TGs. If a participant had more than one lipid profile reading during the study period, only the first reading was collected. The LDL-C was calculated using the Friedewald formula.

Lipid profile components were categorized into four groups:

LDL-C: <2.6, 2.6 to 3.09, 3.1 to 3.59 and ≥3.6 mmol/L.

TC: <4.5, 4.5 to 5.09, 5.1 to 5.69 and ≥5.7 mmol/L.

HDL-C: <1.1, 1.1 to 1.39, 1.4 to 1.59 and ≥1.6 mmol/L.

TG: <0.9, 0.9 to 1.2, 1.21 to 1.69 and ≥1.7 mmol/L.

Assessment of confounders

The confounders considered included body mass index (BMI), statin use, diabetes mellitus (DM), and hypertension (HTN). BMI was categorized as underweight for BMI < 18.5 kg/m2, normal weight for BMI of 18.5-24.9 kg/m2, overweight for BMI of 25-29.9 kg/m2, and obese for BMI > 30 kg/m2). The use of statins was determined electronically by reviewing patient prescriptions. The presence of DM was based on an HbA1C level of ≥6.5%. The presence of HTN was based on multiple charted blood pressure readings of >140/90 mmHg.

Endpoints

Death and cardiovascular outcomes were determined electronically using the patient’s electronic health records in April 2022. The cardiovascular outcomes collected included IHD, heart failure (HF), and stroke.

Statistical analysis

Statistical analysis of the data was done using IBM SPSS Statistics for Windows, Version 20 (Released 2011; IBM Corp., Armonk, New York, United States). Descriptive statistics were reported as the mean and standard deviation for continuous variables, and the categorical variables were summarized as frequencies and percentages. Continuous variables were compared using an independent t-test, while categorical variables were compared using the chi-squared and Fisher’s exact tests. Cox regression was used to estimate the hazard ratio after adjusting for the other variables.

## Results

A total of 670 participants were reviewed. Of those, 485 participants met our inclusion criteria, the mean age was 69.2 ± 6.3 years, and 55.7% of them were female. During the study period, a total of 115 (23.7%) individuals died. The mean follow-up period was 12 years.

Table 1 summarizes the baseline characteristics of the participants based on mortality status. There was no significant difference between alive and deceased participants with regard to the percentage of females, obesity rate, presence of HTN, and the number of people on statins. Factors associated with shorter survival included older age, low HDL-C (<1.1 mmol/L), DM, stroke, and HF.

**Table 1:** Baseline characteristics of the 485 study participants based on mortality status presented as mean ± standard deviation or number (percentage). BMI: Body mass index, TC: total cholesterol, LDL-C: low-density lipoprotein cholesterol, HDL-C: high-density lipoprotein cholesterol, TG: triglyceride

Variables	Alive (n=370)	All-Cause Death (n=115)	P value
Age (years)	68.0±5.4	73.3±7.2	<0.0001
Female gender	207 (55.9)	63 (54.8)	0.826
BMI			0.008
<18.5 kg/m2	0 (0)	2 (1.9)	
18.5-24.9 kg/m2	38 (10.4)	18 (16.7)	
25-29.9 kg/m2	132 (36)	27 (25)	
≳30 kg/m2	197 (53.7)	61 (56.5)	
Hypertension	233 (63)	83 (72.2)	0.070
Diabetes mellitus	245 (66.2)	89 (77.4)	0.024
TC			0.987
<4.5 mmol/L	200 (54.1)	62 (53.9)	
4.5-5 mmol/L	78 (21.1)	23 (20.0)	
5.1-5.69 mmol/L	60 (16.2)	19 (16.5)	
≥5.7 mmol/L	32 (8.6)	11 (9.6)	
LDL-C			0.520
<2.6 mmol/L	155 (41.9)	57 (49.6)	
2.6-3.09 mmol/L	90 (24.3)	26 (22.6)	
3.1-3.59 mmol/L	56 (15.1)	14 (12.2)	
>3.59 mmol/L	69 (18.6)	18 (15.7)	
HDL-C			0.009
<1.1 mmol/L	65 (17.6)	36 (31.3)	
1.1-1.39 mmol/L	138 (37.3)	38 (33.0)	
1.4-1.59 mmol/L	84 (22.7)	16 (13.9)	
≥1.6 mmol/L	83 (22.4)	25 (21.7)	
TG			0.182
<0.9 mmol/L	46 (12.4)	15 (13.0)	
0.9-1.2 mmol/L	135 (36.5)	30 (26.1)	
1.21-1.69 mmol/L	87 (23.5)	29 (25.2)	
≥1.7 mmol/L	102 (27.6)	41 (35.7)	
Ischemic heart disease	76 (20.5)	30 (26.1)	0.209
Stroke	43 (11.6)	38 (33.0)	<0.0001
Heart failure	33 (8.9)	40 (34.8)	<0.0001
Statins	321 (86.8)	95 (82.6)	0.266

**Figure 1:**
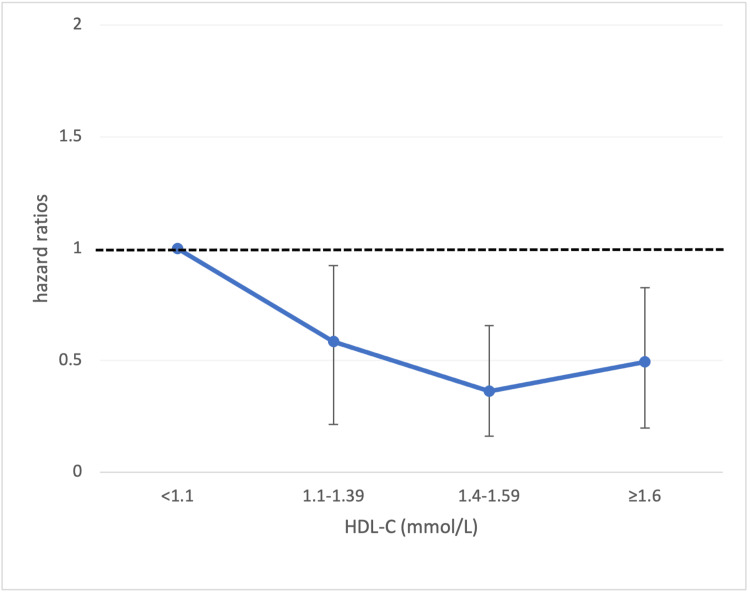
The relationship between HDL-C and all-cause mortality. Adjusted hazard ratios in the second, third, and fourth quartiles compared with the ﬁrst quartile. HDL-C: high-density lipoprotein cholesterol

Figure 1 shows the relationship between HDL-C and all-cause mortality. The multivariable hazard ratios (95% CI) for all-cause mortality were 0.585 (0.371-0.924), 0.363 (0.201-0.655), and 0.494 (0.296-0.825) for the 2nd, 3rd, and 4th quartiles of HDL-C, respectively, when compared to the first quartile, after adjusting for age and DM. TG, LDL-C, and TC levels were not associated with all-cause mortality. Regarding cardiovascular outcomes, low HDL-C (<1.1 mmol/L) was associated with an increased risk of having IHD (adjusted hazard ratio 3.2 (95% CI 1.6-6.2)), and high TC (≥5.7 mmol/L) was associated with an increased risk of having HF (adjusted hazard ratio 2.1 (95% CI 1.1-4.0)).

## Discussion

Dyslipidemia is strongly associated with CVD and mortality, but this association is complicated in the older population. In our study, we explored the relationship between levels of plasma lipids, all-cause mortality, and cardiovascular outcomes. Our findings showed that low HDL-C was significantly associated with a higher risk for all-cause mortality, which is in line with the literature. A Taiwanese cohort study found that low HDL-C (< 1.1 mmol/l) was significantly associated with a high risk for all-cause mortality in people older than 65 years [5]. Low HDL-C was also linked to higher mortality in elderly subjects from a community-based cohort in the United States [11]. Other studies show a similar link [8-10,19], which is likely related to the loss of the well-known protective antioxidative, antithrombotic, and anti-inflammatory effects of HDL-C [25].

Our study did not show a significant association of low TC, LDL-C, or TG levels with all-cause mortality. A recent meta-analysis reviewed the association between LDL-C and mortality in the elderly population and included 19 studies. Of these, four studies similarly found no association, while the rest of the studies showed an inverse relationship with all-cause mortality. For cardiovascular mortality, seven studies showed no association, and only two studies had an inverse association [6].

Regarding TC, there is strong evidence that low levels are associated with higher mortality, particularly all-cause mortality in the older population [3-5]. However, this association is typically attributed to the fact that older people with low TC are linked to having more frailty, nutritional deficiencies, and comorbidities like cancer and other terminal diseases [26]. In our study, we tried to eliminate these factors by excluding subjects who died within one year of follow-up.

Globally, CVD imposes the most significant disease burden among people aged 60 years and older [27]. We investigated the association between the plasma lipid profile and cardiovascular outcomes (IHD, CHF, and stroke). Our results showed that low HDL-C was significantly associated with an increased risk of IHD, similar to what other studies targeting the elderly have reported [8-10, 19]. Furthermore, our results showed a significant association between high TC and CHF. A previous study on the association between TC and cardiovascular outcomes showed a similar link with a higher risk in subjects with high TC [22].

The role of low HDL-C levels as a predictor of cardiovascular morbimortality and all-cause mortality in the older population has been proven in studies done in Western countries and East Asia [5,8-11,19]. Yet global lipid management guidelines mainly target LDL-C and not HDL-C, including those of the American College of Cardiology/American Heart Association (ACC/AHA) [28] and the National Institute for Health and Care Excellence (NICE) [29]. Additionally, these guidelines do not emphasize the elderly and primarily target the middle age group.

To the best of our knowledge, this study is the first in Saudi Arabia and the region to investigate the association of plasma lipid concentrations with all-cause mortality and cardiovascular outcomes in an older population. This is particularly important as the country is following the global increase in the aging population, with the proportion of those aged 60 years and above projected to increase to 9.5% and 18.4% in 2035 and 2050, respectively [30].

Limitations

Due to the nature of this study design, reverse causality may be a concern, as the direction of the relationship between exposure and outcome may be unclear in retrospective studies. Also, we could not identify cause-specific mortality due to the lack of current infrastructure for retrieving data from the death registry to specify the cause of death. Furthermore, a larger prospective cohort study will be necessary to obtain more robust data regarding the older Saudi population.

## Conclusions

Our study showed that in patients aged 60 years and older, low HDL-C (<1.1 mmol/L) was associated with a higher risk of all-cause mortality and IHD. High TC was associated with an increased risk of having CHF. No significant association was found for LDL-C, TC, and TG with increased risk for all-cause mortality. With current dyslipidemia management guidelines targeting LDL-C, it is safe to argue that more focus on targeting HDL-C is needed. Additionally, future research should explore the causes and risk factors of low HDL-C in the elderly.
